# Conformal Engineering of Both Electrodes Toward High‐Performance Flexible Quasi‐Solid‐State Zn‐Ion Micro‐Supercapacitors

**DOI:** 10.1002/advs.202308021

**Published:** 2024-04-01

**Authors:** Yaopeng Wu, Wei Yuan, Pei Wang, Xuyang Wu, Jinghong Chen, Yu Shi, Qianyi Ma, Dan Luo, Zhongwei Chen, Aiping Yu

**Affiliations:** ^1^ School of Mechanical and Automotive Engineering South China University of Technology Guangzhou 510640 China; ^2^ Department of Chemical Engineering University of Waterloo Waterloo N2L 3G1 Canada

**Keywords:** electrode engineering, energy storage devices, flexibility, patternable electrode, Zn‐ion micro‐supercapacitors

## Abstract

The severe Zn‐dendrite growth and insufficient carbon‐based cathode performance are two critical issues that hinder the practical applications of flexible Zn‐ion micro‐ssupercapacitors (FZCs). Herein, a self‐adaptive electrode design concept of the synchronous improvement on both the cathode and anode is proposed to enhance the overall performance of FZCs. Polypyrrole doped with anti‐expansion graphene oxide and acrylamide (PPy/GO‐AM) on the cathode side can exhibit remarkable electrochemical performance, including decent capacitance and cycling stability, as well as exceptional mechanical properties. Meanwhile, a robust protective polymeric layer containing reduced graphene oxide and polyacrylamide is self‐assembled onto the Zn surface (rGO/PAM@Zn) at the anode side, by which the “tip effect” of Zn small protuberance can be effectively alleviated, the Zn‐ion distribution homogenized, and dendrite growth restricted. Benefiting from these advantages, the FZCs deliver an excellent specific capacitance of 125 mF cm^−2^ (125 F cm^−3^) at 1 mA cm^−2^, along with a maximum energy density of 44.4 µWh cm^−2^, and outstanding long‐term durability with 90.3% capacitance remained after 5000 cycles. This conformal electrode design strategy is believed to enlighten the practical design of high‐performance in‐plane flexible Zn‐based electrochemical energy storage devices (EESDs) by simultaneously tackling the challenges faced by Zn anodes and capacitance‐type cathodes.

## Graphical abstract

A self‐adaptive electrode design concept of synchronous improvement on both the cathode and anode is proposed for flexible Zn‐ion micro‐supercapacitors (FZCs). During the fabrication of the doped polypyrrole (PPy) cathode, a robust protective layer containing reduced graphene oxide (rGO) and polyacrylamide (PAM) is self‐assembled onto the Zn anode surface. This design enhances the overall performance of the FZCs.

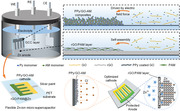

## 1 Introduction

The development trends for modern electrochemical energy storage devices (EESDs) encompass characteristics such as planar geometry, flexibility, miniaturization, lightweight design, appealing energy/power density, and ease of industrial scalability.^[^ ^1^, ^2^, ^3^, ^4^, ^5^, ^6^ ^]^ Flexible Zn‐ion micro‐supercapacitors (FZCs), assembled with a metallic Zn anode and a capacitive‐type cathode integrated onto a flexible substrate, have been widely investigated due to their superior energy density compared to traditional capacitors and remarkable power density relative to Li‐ion batteries.^[^ ^7^, ^8^ ^]^ Despite significant progress, the practical realization of FZCs still confronts dual challenges rooted in material science and fabrication technology. Regarding the capacitive‐type cathode, it is in a dilemma to meet the requirements of high capacitance and long‐term cyclability. The limited capacitance of the cathode leads to an undesirable energy density, and it is getting even worse when it comes to carbon‐based materials. Pseudocapacitive materials typically offer higher capacitance by facilitating rapid faradaic reactions near the electrode surface. However, they usually suffer from structural degradation during the charge/discharge cycles, impacting long‐term cycling performance.^[^ ^9^ ^]^ In terms of the Zn anode, the growth of Zn dendrites is a consequence of non‐uniform Zn plating/stripping stemming from uneven distribution of Zn^2+^ ions and electric field gradients. This phenomenon leads to a series of detrimental effects, including increased polarization, low coulombic efficiency (CE), the formation of “dead” Zn, hydrogen evolution, and ultimately, device failure induced by internal short circuits.^[^ ^10^, ^11^ ^]^

Hitherto, to enhance the overall performance of Zn‐based EESD, considerable efforts have been made through the introduction of optimized capacitive‐type cathode (e.g., designing porous structures,^[^ ^12^, ^13^ ^]^ surficial functional groups,^[^ ^14^, ^15^ ^]^ and doped materials^[^ ^16^, ^17^ ^]^) and modified metallic Zn anode (e.g., designing protective layers,^[^ ^18^, ^19^ ^]^ structural anodes,^[^ ^20^, ^21^ ^]^ and Zn‐based alloys).^[^ ^22^, ^23^ ^]^ Nevertheless, the vast majority of these strategies are obsessively focusing on either improving cathode capacitance or suppressing Zn‐dendrite growth. Scarcely, few studies have demonstrated scalable design strategies that enhance both cathodic and anodic performance in a single Zn‐based EESD to match the performance of both electrodes.^[^ ^24^, ^25^ ^]^ Therefore, it is of significance to develop a high‐performance flexible Zn‐based EESD through simultaneous enhancement on both Zn anode and capacitive‐type cathode. Additionally, struggle remains in the integration of asymmetric configurations in planar flexible devices with diverse electrode materials. In this respect, a few fabrication techniques for in‐plane FZCs, including mask‐assisted filtration,^[^ ^26^ ^]^ photolithography,^[^ ^27^ ^]^ laser carving,^[^ ^28^ ^]^ screen printing, and direct ink writing,^[^ ^29^, ^30^ ^]^ have been proposed recently. These approaches have achieved certain enhancements in either the metallic Zn anode or the capacitive‐type cathode. Still, there is an urgent need to diversify manufacturing strategies for planar custom‐shaped FZCs compatible with synchronous performance enhancement of both metallic Zn anode and capacitive‐type cathode.

Toward this end, we developed a self‐adaptive design strategy for high‐performance FZCs by simultaneously forming cathodic material on the cathode and anodic protection layer on Zn surface in a single‐step process. First, the planar interdigitated pattern of FZCs is predefined and prepared by facile mask‐assisted dry transfer technique. For the capacitive‐type cathode, doped PPy composites (PPy/GO‐AM) with superior cyclability and mechanical performance are prepared through the electro‐polymerization of pyrrole (Py) in the presence of graphene oxide (GO) and acrylamide (AM). Notably, during the preparation of the cathode, a polymeric composite layer consisting of reduced GO (rGO) and polyacrylamide (PAM) self‐assembles and deposits onto the Zn surface (rGO/PAM@Zn), resulting in a robust conformal protective layer. This layer effectively homogenizes Zn‐ion flux and inhibits Zn dendrite growth, leading to stable Zn stripping/plating behavior. Consequently, the protected Zn exhibits excellent cyclability for 1000 h at 1 mA cm^−2^ and 1 mAh cm^−2^. The as‐obtained FZCs with these two optimized electrodes present an outstanding specific capacitance of 125 mF cm^−2^ (125 F cm^−3^) at 1 mA cm^−2^, high energy density of 44.4 µWh cm^−2^ at a power density of 0.8 mW cm^−2^, and ultra‐stable cycling durability with 90.3% capacitance remaining after 5000 cycles at a large scan rate of 100 mV s^−1^, surpassing many of the previously reported EESDs. More importantly, the self‐adaptive optimization strategy also endows FZCs with favorable mechanical properties. In short, we ensure the excellent electrochemical and mechanical performance of FZCs by producing high‐capacity PPy cathode materials and high‐stability Zn anode materials through a one‐step optimization strategy. The proposed feasible manufacturing process is of scalability and designability, providing practical guidance for the fabrication of high‐performance planar Zn‐based EESDs.

## 2 Result and Discussion

### 2.1 Fabrication of FZCs

**Figure 1:**
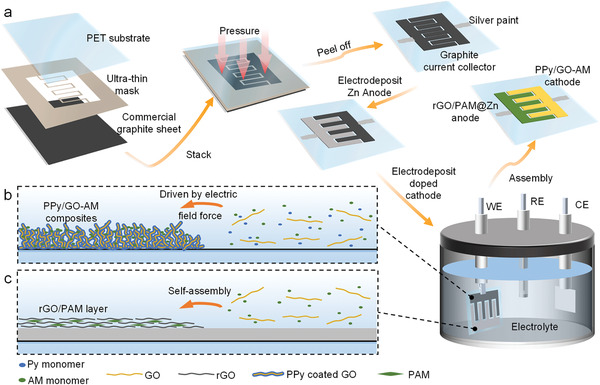
Schematic illustration of the fabrication of FZCs with optimized electrodes. a) Preparation process of FZCs. b) Deposited PPy/GO‐AM composites on GCC under electric field force. c) Sefl‐assembled rGO/PAM layer on GCC.

**Figure** **1** illustrates the complete manufacturing process for planar FZCs. Initially, a mask‐assisted dry transfer technique was employed to create a flexible graphite current collector (GCC) layer with an interdigitated shape on a PET substrate (Figure 1a). An ultra‐thin PET film with a customized shape was utilized as the transfer mask. The interdigital pattern was predefined and manufactured by UV laser engraving (Figure S1, Supporting Information). A commercial graphite sheet with high electric conductivity served as the raw material for the GCC layer. To be specific, the graphite sheet, as‐prepared mask, together with flexible PET transfer film were stacked into a sandwich configuration. After applying pressure and subsequently disassembling the components, the interdigitated GCC layer was transferred and firmly adhered to the upper PET substrate. The outstanding adhesive ability of PET to carry GCC benefits from its strong electrostatic adsorption. This dry transfer technique is flexible and enables the construction of intricate patterns, including our university logo (Figure S2, Supporting Information). After the transfer process, a Zn anode was electroplated on one side of the GCC layer. Subsequently, the as‐resulted sample was immersed in a deliberately prepared electrolyte for the deposition of PPy/GO‐AM electrode on the opposite side of the GCC layer. The electrolyte consists of Py, GO, AM, and *β*‐naphthalene sulfonic acid as an anionic dopant for the electrochemical polymerization of PPy. During the cathode preparation, the electrochemical oxidation and polymerization of Py monomer occurred. Simultaneously, GO in the solution was encapsulated by the PPy polymer, forming a porous composite (PPy/GO) attached to the GCC film under the electric field force. Additionally, AM was also successfully incorporated to enhance the cycling stability and mechanical performance of the cathode (Figure 1b). Significantly, at the side of the Zn anode, GO, acting as an oxidizing agent, spontaneously reacted with Zn in the mild acidic solution, and was reduced to rGO deposited onto the Zn surface. Besides, PAM, as an addition polymer, is readily formed when AM monomers containing C═C double bonds add to each other with the help of initiators. Here, the as‐produced rGO served as an initiator and triggered the polymerization of AM, leading to the formation of a protective rGO/PAM layer for a highly reversible Zn anode (Figure 1c). As presented in Figure S3 (Supporting Information), photographs of different stages in the preparation validate the successful optimization of both the Zn anode and the doped cathode. It is worth mentioning that the introduction of AM enables the uniform deposition of the protective layer. However, without AM additive, deposited pure rGO tends to crack and detach from the Zn surface, which is unable to form a stable film on the surface (Figure S4, Supporting Information). The material deformation has resulted from the inner microstructural stress caused by the shrinkage of rGO during the drying process. Afterward, the as‐fabricated sample was rinsed with water and ethanol to remove residual chemicals, including GO, AM, and unreacted Py remaining on the rGO/PAM protective layer. Finally, a planar FZC was obtained after the drop‐casting of gelatin electrolyte and device dehumidification.

### 2.2 Material Characterization and Electrochemical Performance of PPy/GO‐AM Cathode

**Figure 2:**
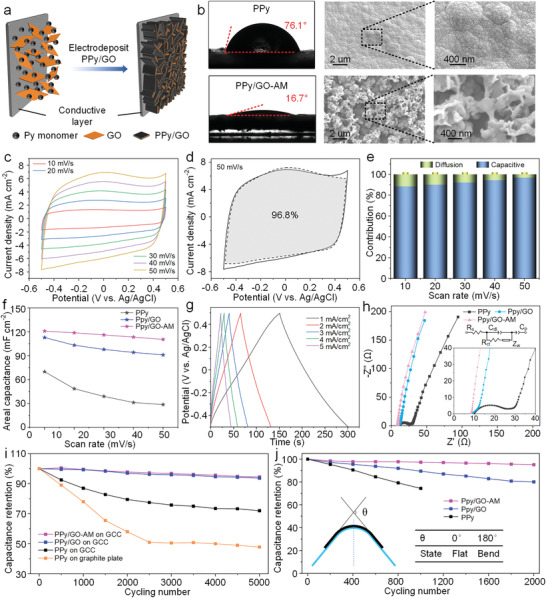
a) Schematic illustration of the electrodeposition of PPy/GO. b) Wetting ability and SEM images of PPy (top) and PPy/GO‐AM (bottom). c) CV curves of PPy/GO‐AM obtained at scan rates of 10–50 mV s^−1^. d) Capacitive contribution of PPy/GO‐AM at scan rate of 50 mV s^−1^. e) Capacitive ratio of PPy/GO‐AM at stepwise scan rate. f) Areal capacitance of PPy, PPy/GO, and PPy/GO‐AM at different scan rates. g) GCD curves of PPy/GO‐AM at different current densities. h) EIS plot of PPy, PPy/GO, and PPy/GO‐AM, and equivalent circuit model. i) Cycling performance at a scan rate of 50 mV s^−1^. j) Bending stability recorded at 20 mV s^−1^ after every 200 bending cycles with a total of 2000 cycles.

To investigate the material properties of the cathode, PPy/GO‐AM composite is fabricated and deposited onto the flexible GCC layer. The schematic illustration of the electrochemical deposition of Py in the presence of GO is shown in **Figure** **2**a. Under the influence of an electric field, unstable Py radical cations are formed at the graphite surface due to the loss of electrons from Py. Subsequently, Py dimers are generated by combining the radical cations and another Py monomer. Following the chain growth steps, PPy macromolecular chains can be obtained and coated on the surface of GO, forming PPy/GO composite conformed on the graphite sheet. Fourier‐transform infrared (FTIR) spectra are conducted to validate the formation of the PPy/GO composite (Figure S5, Supporting Information). The featured peaks of the oxygen‐contained functional group of GO are found in the spectrum. Specifically, the peaks at 3418, 1728, 1600, and 1243 cm^−1^ correspond to the O─H, C═O, C─C, and C─O stretching vibrations, respectively.^[^ ^31^ ^]^ The spectrum of PPy shows the characteristic peaks related to the Py ring, such as the carbon skeleton vibration at 1551 cm^−1^, the stretching vibration of C─N at 1463 cm^−1^, and the deformation vibration of C─H at 1046 cm^−1^.^[^ ^32^ ^]^ The FTIR curves of pure PPy and PPy/GO composite present similar characteristics to a large extent, demonstrating the presence of PPy in the doped composite. Importantly, compared to GO, the C═O stretching peak of the PPy/GO composite shifts to a lower wavenumber of 1697 cm^−1^, which indicates the formation of hydrogen bonds between GO (─C═O group) and PPy (─NH group).^[^ ^33^ ^]^ In addition, as presented in Raman spectra (Figure S6, Supporting Information), the red shift of the G band in the PPy/GO composite also suggests the successful preparation of the hybrid polymer. The PPy/GO‐AM composite is obtained through doping AM into PPy/GO composite. The conspicuous peaks of the amide group (─C═O stretching at 1679 cm^−1^, ─NH_2_ bending at 1562 cm^−1^) are observed in the FTIR curve of the PPy/GO‐AM composite (Figure S7, Supporting Information), indicating the incorporation of AM.^[^ ^34^ ^]^ The wettability of these samples is assessed by contact angle tests. Upon the introduction of AM, the contact angle of the PPy/GO‐AM composite with 2 µL of 1 m KCl drop dramatically decreases to 16.7°, which is significantly smaller than that of pure PPy (76.1°) (Figure 2b). The excellent infiltration of PPy/GO‐AM is attributed to the porous structure and the hydrophilicity of the amide polar group. The microstructure and inner morphology are examined by scanning electron microscope (SEM) measurements. The PPy/GO composite presents a 3D vertical structure (Figure S8, Supporting Information), while the pure PPy shows a flat and dense feature (Figure 2b). Notably, in addition to the porous structure, the PPy/GO‐AM composite also possesses interconnected features, which are believed to confer improved capacitance and mechanical properties.

The electrochemical performance of PPy, PPy/GO, and PPy/GO‐AM was investigated in a three‐electrode system with a 1 m KCl electrolyte. As displayed in Figure 2c, the C–V curves at different scanning rates all show deviated rectangular shapes, suggesting two types of energy storage: the electrostatic adsorption of electrolyte ions on the polarized electrode surface; the pseudo‐capacitance generated by insertion/extraction of electrolyte ions in PPy. From the C*–*V curves of pure PPy and doped composites, it can be concluded that all the fabricated materials possess the same energy storage mechanism (Figure 2c; Figure S9, Supporting Information). Doped electrodes like PPy/GO and PPy/GO‐AM delivered higher capacitance than pure PPy, as evidenced by the larger enclosed area of C–V curves (Figure S10, Supporting Information). As shown in Figure 2d, at the scan rate of 50 mV s^−1^, the capacitance contribution of PPy/GO‐AM reached up to 96.8%, higher than that of pure PPy and PPy/GO (Figure S11 and Table S1, Supporting Information). The calculated data reveal that the charging/discharging process exhibits a significant capacitance‐controlled behavior, including surface electro‐sorption, near‐surface, and intercalation pseudo‐capacitance. The ratio of capacitive contributions is gradually raised as the scan rate increases (Figure 2e), indicating that the lack of time renders ions to mainly participate in surface/near surface reactions. The satisfactory capacitive performance of PPy/GO‐AM is attributed to plenty of accessible ion channels arising from its porous structure and spontaneous adsorption of electrolyte ions by hydrophilic amide groups.^[^ ^35^ ^]^ The exceptional rate performance of PPy/GO‐AM is proved by recorded capacitances under different scan rates (Figure 2f). The better rate performance can be ascribed to the superb reaction reversibility and structural stability of PPy/GO‐AM. The excellent reversibility is confirmed by the favorable symmetry of the triangular shape shown in the GCD curves at different current densities (Figure 2g; Figure S12, Supporting Information). Compared with doped electrodes, pure PPy electrode has a distinct IR drop, demonstrating limited electrochemical reversibility and large internal resistance (Figure S13, Supporting Information). As shown in the middle‐frequency range of the electrochemical impedance spectroscopy (EIS) plot (Figure 2h), it can be seen that the migration rate of ions from the electrolyte to the doped electrodes is much higher than that of the pure PPy electrode. The exceptional results come from the excellent wettability of doped PPy. Besides, the EIS curve of PPy/GO‐AM exhibits the largest slope in the low‐frequency range, suggesting excellent capacitance properties. The cyclability of PPy/GO‐AM has also been measured, and it shows an admirable capacitance retention of up to 94.5% after 5000 cycles even at a relatively high scan rate of 50 mV s^−1^ (Figure 2i). However, PPy showed limited capacitance retention after cycling, with 72% of the original capacitance remaining. Notably, compared with PPy on a flexible GCC thin layer, PPy deposited on a bulk graphite plate exhibited a more pronounced degradation in capacitance (47.8% of retention). This result proves that the mechanical flexibility of the GCC substrate ensures the enhanced cyclability of PPy by accommodating the large volumetric expansion during the charging/discharging process.^[^ ^36^ ^]^ Although the introduction of AM doping shows a limited effect on long‐term cyclability, it significantly boosts the mechanical properties against macroscopic deformation. The bending stability of PPy/GO‐AM electrode was performed under repeatedly flat/bending cycle conditions. The capacitance data were recorded and calculated after every 200 cycles out of a total of 2000 bending cycles. Impressively, the PPy/GO‐AM exhibited high capacitance retention of 95% after repeated bending experiments (Figure 2j). The excellent mechanical performance can be attributed to the interconnected structure of PPy/GO‐AM composites devoted to the addition of AM.

### 2.3 Formation Mechanism and Characterization of rGO/PAM Protective Layer

**Figure 3:**
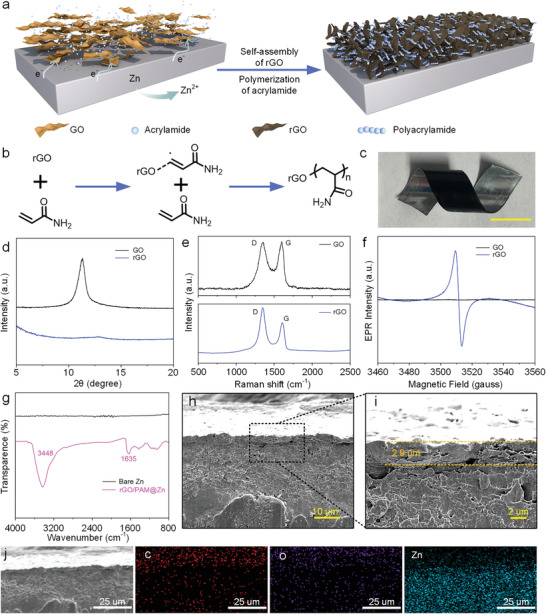
a) Schematic illustration of reduction of GO and polymerization of AM. b) An unpaired electron from the rGO reacts with the *π*‐bond of the vinyl monomer. c) Photograph of Zn foil deposited with rGO/PAM protective layer at a bent state. d) XRD and e) Raman spectra of GO and rGO. f) EPR signals of GO and rGO. g) FTIR spectra of bare Zn and rGO/PAM@Zn. h) SEM image (cross‐sectional view) of rGO/PAM@Zn and i) corresponding enlarged image. j) SEM images of rGO/PAM@Zn and corresponding elemental distribution of EDS mapping.

Zn, known as a mild reactive metal with strong reductivity, exhibits a remarkable capability to effectively reduce GO in an aqueous medium. In an acidic solution, Zn loses electrons, forcing the positively charged GO into contact with the Zn surface. As GO receives electrons, oxygen‐containing groups coordinated with hydrogen ions detach from the GO surface, leading to the dehydration and reduction of GO. Interestingly, once AM is introduced into the above system, not only a 3D rGO network is formed and intimately contacted on the Zn surface, but also the polymerization of AM can be observed. The self‐assembly of rGO and the polymerization of AM are illustrated in **Figure** **3**a. The free radical polymerization of AM and the impact of the rGO will be discussed in this section. It's worth mentioning that we focus more on the thorough investigation of the formation mechanism of the rGO/PAM layer, rather than the capacity offered by the protective structure. This choice is due to the understanding that the capacity of the anode is mainly attributed to the reversible stripping and deposition of Zn.

The formation mechanism of the conformal rGO/PAM protective layer can be explained as below. First, oxygen‐containing functional groups bonded to the carbon atoms were removed in the reduction of GO, leaving behind considerable oxygen vacancy defects in the original positions of carbon skeleton. Due to the breaking of C─O bonds, the carbon atoms around oxygen vacancies would generate unpaired electrons, which has also been described as ′graphene free radical2 in some reported research.^[^ ^37^, ^38^, ^39^ ^]^ Afterward, the unpaired electrons reacted with AM‐monomer *π* bonds, triggering the polymerization through covalent‐bond formation between rGO and AM‐monomer carbon atoms (Figure 3b). The continuously generated free radical endowed the chain propagation of AM, finally leading to a cross‐linked rGO/PAM layer on Zn. As shown in Figure 3c, benefiting from the robust adhesion of the polymer material, the conformal rGO/PAM layer stuck tightly to the Zn surface even in a bending state. The effective reduction of GO caused by Zn was evidenced by the X‐ray diffraction (XRD) of the reaction product (Figure 3d). The disappeared diffraction peak (2*θ* = 11.3°) in the XRD pattern of rGO demonstrated its smaller interlayer spacing than that of GO, which resulted from the removal of oxygen‐containing groups. The Raman spectrum of GO displayed two predominant *D* and *G* bands with peaks at 1351 and 1590 cm^−2^, respectively (Figure 3e). The intensity ratio of *D* and *G* bands of rGO (I_D_/I_G_ = 1.43) was much higher than that of GO (I_D_/I_G_ = 1.08), indicating the alteration of graphene planar structure, along with a high quantity of structural defects.^[^ ^40^ ^]^ Electron paramagnetic resonance (EPR) measurement was conducted to directly examine the unpaired electrons caused by oxygen vacancies (Figure 3f). It can be seen that, compared with GO, the rGO sample shows a prominent signal in EPR, confirming the presence of a large number of oxygen vacancies on the carbon backbone, or so‐called graphene free radical. The as‐resulted radical initiates the in situ polymerization of AM. As shown in the FTIR spectra (Figure 3g), the typical bands at 1635 and 3448 cm^−1^ are attributed to the stretching vibration of N─H, and the C═O stretching in the amide group (─CONH_2_), respectively.^[^ ^41^ ^]^ Besides, the elimination of the characteristic C═C peak indicates the complete polymerization of AM to PAM (Figure S14, Supporting Information).

The SEM images validate the stacked structure of the deposited rGO and the firm contact between rGO/PAM and Zn (Figure 3h,i). The EDS elemental mapping demonstrates that the rGO/PAM protective layer has been successfully deposited on the Zn surface (Figure 3j). The average thickness of the rGO/PAM layer is evaluated to be ≈2.9 µm. Notably, with the addition of AM, the reduction reaction rate of GO and Zn was suppressed to a certain degree, due to the generated PAM polymer layer (Figure S15, Supporting Information). It is worth mentioning that though Py monomer was added to the electrolyte during the fabrication of the flexible device, neither PPy polymer nor Py monomer was observed in the rGO/PAM composites after being rinsed by water and ethanol several times. This was evidenced by the combined results of SEM images and FTIR spectra, where there is no obvious difference in the as‐deposited rGO/PAM composites in the presence or absence of the Py monomer (Figures S15 and S16, Supporting Information).

### 2.4 Electrochemical Performance of rGO/PAM@Zn Anode

**Figure 4:**
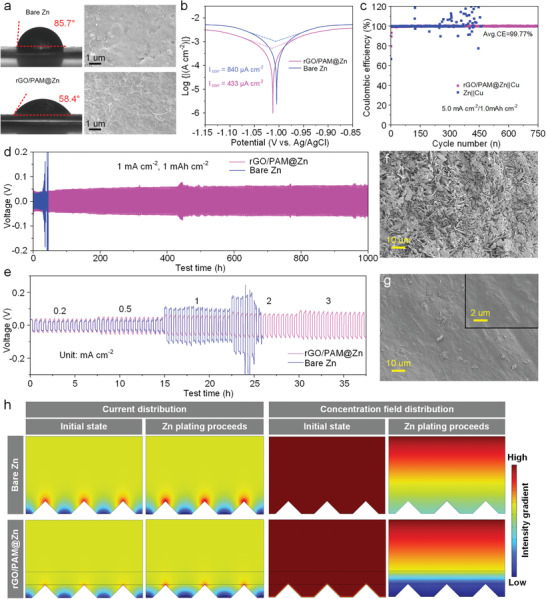
a) Wetting ability and top‐view of SEM images of bare Zn (top) and rGO/PAM@Zn (bottom). b) Tafel plots presenting the corrosion for bare Zn and rGO/PAM@Zn. c) CE tests of Zn||Cu and rGO/PAM@Zn||Cu at a current density of 5 mA cm^−2^. d) Long‐term cycling performance of symmetrical bare Zn and rGO/PAM@Zn cell at a current density of 1 mA cm^−2^. e) Rate performance of symmetrical bare Zn and rGO/PAM@Zn cell at various current densities form 0.2 to 3 mA cm^−2^. f) Top view of SEM image of bare Zn after 50 cycles at 1 mA cm^−2^. g) Top view of SEM image of rGO/PAM@Zn after 250 cycles at 1 mA cm^−2^. h) Theoretical simulation of current and Zn^2+^ ions concentration field distribution for bare Zn and rGO/PAM@Zn.

First, the hydrophilicity of different electrodes was measured by testing the contact angle between the electrolyte (2 µL of 1 m ZnSO_4_) and the electrode surface. As shown in **Figure** **4**a, the wetting angle test results indicate that rGO/PAM@Zn possesses enhanced wetting ability (58.4°) compared to bare Zn (85.7°). The improved wetting performance is mainly ascribed to the microporous structure of the deposited rGO and the excellent water absorption capability of amide polar groups. The superior hydrophilicity is believed to promote homogeneous Zn^2+^ nucleation and deposition behavior.^[^ ^42^ ^]^ Besides, the anticorrosive property of rGO/PAM layer was demonstrated by Tafel plots. As illustrated in Figure 4b, the corrosion current density of rGO/PAM@Zn sample is estimated to be ≈433 µA cm^−2^, lower than that of bare Zn (≈840 µA cm^−2^), indicating the better corrosion resistance performance of rGO/PAM@Zn with the coated conformal rGO/PAM protective layer. In addition, the corrosion potential of rGO/PAM@Zn is slightly positively shifted, suggesting its reduced corrosion tendency.

To investigate the reversibility of rGO/PAM@Zn anode in the stripping/plating process, the CE experiment was carried out by testing rGO/PAM@Zn||Cu asymmetric cell in a 2 m ZnSO_4_ electrolyte. As seen in Figure 4c and Figure S17 (Supporting Information), rGO/PAM@Zn||Cu presented a stable Zn plating/stripping process for 900 cycles and 750 cycles at relatively high current densities of 2 and 5 mA cm^−2^, respectively. The corresponding average CE was as high as 99.68% and 99.77%. Unfortunately, the Zn||Cu cells all failed after ≈300 cycles at different current densities. The electrochemical stability of the protected Zn anode was also evaluated by continued Zn plating/stripping in symmetric cells. The bare Zn suddenly went short circuit after 280 h cycling at a current density/loading capacity of 0.2 mA cm^−2^ and 0.2 mA h cm^−2^ (Figure S18, Supporting Information). In contrast, the rGO/PAM@Zn showed a stable polarization and long cyclability of 1300 h. Furthermore, compared with bare Zn, rGO/PAM@Zn exhibited superior electrochemical stability for 1000 h when the current density/loading capacity was increased to 1 mA cm^−2^ and 1 mA h cm^−2^ (Figure 4d). As listed in Table S2 (Supporting Information), the excellent reversibility is better than that reported in many other researches. The rate performance of rGO/PAM@Zn was conducted under different current densities, where the protected Zn showed great rate stability, while the high polarization voltage was observed in bare Zn at 2 mA cm^−2^ (Figure 4e). The failure of bare Zn was mainly attributed to the short circuit of symmetric cells, which resulted from the massive growth of Zn dendrites piercing the separator. As displayed in Figure 4f, the uneven Zn accumulation was evidenced by the SEM image of the cycled bare Zn. Notably, the rGO/PAM@Zn still showed a smooth surface after cycling for 500 h (1 mA cm^−2^ and 1 mA h cm^−2^) (Figure 4g). Theoretical simulation was also performed by COMSOL to support the uniform deposition of Zn at rGO/PAM@Zn (Figure 4h). For bare Zn, the current distribution shows a compact intensity gradient during the plating process (Figure 4h, (top)), which leads to preferential deposition of Zn on top of small protuberances under the tip effect. The sustained deposition consuming a large amount of Zn^2+^ ions causes a local inhomogeneous distribution of Zn^2+^ ions concentration (Figure 4i (top)). The uneven distribution of Zn^2+^ ions concentration induces irregular Zn deposition and worse electric field distribution. Repeatedly, spiny Zn dendrites evolve and densely cover on the Zn surface. In the case of rGO/PAM@Zn, owing to the charge redistribution effect and the isolation effect of the polymer,^[^ ^43^ ^]^ the introduction of conformal rGO/PAM layer uniformizes the current distribution, especially near the top of the small Zn protuberances (Figure 4h, (bottom)). Significantly, benefiting from the regulation of the adaptive rGO/PAM layer, a more homogeneous Zn^2+^ ions concentration is generated at the interface between Zn and the electrolyte (Figure 4i (bottom)). The reduced polarization of Zn^2+^ ions concentration near the electrode enables a more uniform Zn deposition, therefore leading to a flat and smooth surface.

### 2.5 Performance of FZCs

To verify the practicability of the conformal optimization strategy, FZCs were fabricated, and the electrochemical performances were characterized. The rGO/PAM protective layer was observed to be assembled on a flexible Zn anode in the cross‐section view of SEM image (Figure S19, Supporting Information). The corresponding elemental distribution of EDS mapping confirms the sandwich‐like structure consisting of PET substrate, GCC layer, Zn anode, and protective rGO/PAM layer. The thickness of both PPy/GO‐AM cathode and rGO/PAM@Zn anode is estimated to be 10 µm from the cross‐section view of the SEM image (Figure S20, Supporting Information). The loading of the Zn anode is ≈5 mAh cm^−2^ (6.09 mg cm^−2^). Significantly, given the negligible weight of the active materials in such a thin film, it is more reasonable to use areal capacitance and volumetric capacitance for a comprehensive evaluation of the electrochemical performance.

**Figure 5:**
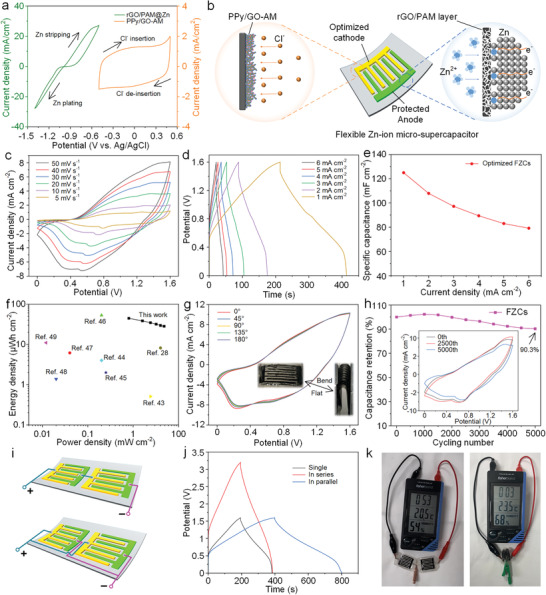
a) C–V curves at 10 mV s^−1^ showing Zn plating/stripping at rGO/PAM@Zn anode, and Cl^−^ insertion/extraction at PPy/GO‐AM cathode. b) Schematic illustration of electrochemical reaction during the charging/discharging process of FZCs. c) CV curves of FZC obtained at scan rates of 5–50 mV s^−1^. d) GCD curves of FZC at different current densities from 1–6 mA cm^−2^. e) Areal capacitance of FZC. f) Ragone plot of our FZC and previously reported flexible planar EESDs. g) C–V curves of FZC under different bending angles from 0° to 180° tested at 50 mV s^−1^. h) Cycling performance of FZC at a scan rate of 100 mV s^−1^. i) Schematic illustration of two FZCs connected in series (top) and in parallel (bottom), and j) corresponding GCD curves (1 mA cm^−2^). k) Photographs of two serial connected FZCs powering a digital monitor.

The electrochemical reaction process of the Zn and the PPy/GO‐AM was analyzed by CV measurement (**Figure** **5**a). At the Zn side, apparent redox peaks appear at −0.6/−1.3 V (vs Ag/AgCl), demonstrating the stripping/plating process of Zn/Zn^2+^.^[^ ^44^ ^]^ In the case of the PPy/GO‐AM, during the cycling process, the electrochemical double layers are formed, along with the insertion/extraction of Cl^−^ into PPy.^[^ ^45^ ^]^ Since there are different potential ranges in Zn and PPy/GO‐AM, FZCs can be assembled based on the Zn anode and doped PPy cathode. The electrochemical reaction during the charging/discharging cycle is schematically presented in Figure 5b. During the charging process, under the effect of electric field force, Zn^2+^ was migrated to the Zn surface and reduced to Zn atoms when receiving electrons. At the same time, the negatively charged Cl^−^ was absorbed onto the cathode and inserted into the PPy/GO‐AM composite, producing both electrical double‐layer capacitance and pseudo‐capacitance.

The electrochemical performance of the protected FZCs was examined by assembling quasi‐solid‐state devices with ZnSO_4_/KCl gelatin electrolyte. The CV measurements were carried out at varied scan rates ranging from 5 to 50 mV s^−1^ (Figure 5c). The CV profiles of FZCs reveal a high potential window of 0–1.6 V. The unique shapes deviated from classical rectangular validate the combined charge storage mechanisms in FZCs. The high reversibility of the charging/discharging process was supported by the triangular‐like GCD curves at different current densities of 1–6 mA cm^−2^ (Figure 5d). Rate performance calculated from the GCD curves demonstrates an excellent specific capacitance of 125 mF cm^−2^ (125 F cm^−3^) at 1 mA cm^−2^ (Figure 5e). The FZCs still maintain a satisfactory capacitance of 80 mF cm^−2^ even at a high current density of 6 mA cm^−2^. As highlighted in the Ragone plot (Figure 5f), the as‐fabricated FZC conveys a maximum areal energy density of 44.4 µWh cm^−2^ at a power density of 0.8 mW cm^−2^, and still remains 28.1 µWh cm^−2^ at a maximum power density of 4.8 mW cm^−2^. It is worth mentioning that only 10 µm of active electrode materials are deposited on the GCC layer. The thickness and mass loading of the active material can be further increased by extending the deposition capacity. Nevertheless, the results of the areal energy/power density are superior to those of the flexible planar EESDs reported in the open literature.^[^ ^29^, ^46^, ^47^, ^48^, ^49^, ^50^, ^51^, ^52^ ^]^ More details on the areal capacitance and energy/power densities of the EESDs are summarized in Table S3 (Supporting Information) for comparison.

To investigate the favorable mechanical flexibility of the FZCs, the electrochemical performance under different bending states has been evaluated by CV measurements. As depicted in Figure 5g, all CV profiles overlapped almost completely, even in the fully folded state, demonstrating the outstanding flexibility and electrochemical stability of the planar FZC. The excellent cyclability of the FZC sample was confirmed by the exceptional capacitance retention of 90.3% after 5000 cycles at a high scan rate of 100 mV s^−1^ (Figure 5h). The outstanding long‐term durability is ascribed to the synchronous reinforcement on both capacitive‐type cathode and Zn anode demonstrated above. An energy storage pack with two FZCs serially connected was fabricated (Figure 5i, top), and the operating voltage window was enlarged to 0–3.2 V (Figure 5j), suggesting the successful integration. Furthermore, when the pack is constructed by two FZCs connected in parallel (Figure 5i, bottom), the discharging time is twice than that of one single FZC, demonstrative of the exceptional uniformity of the as‐prepared FZCs. As a physical demonstration, two samples connected in series can drive a digital monitor, and the device still works even in a highly bent state (Figure 5k). These results evidence that integrated FZCs show appreciable application potential in flexible electronics.

## 3 Conclusion

In summary, we developed a feasible electrode design strategy for high‐performance FZCs through conformal engineering with synchronous improvement on both the cathode and anode. A planar GCC layer with an interdigitated pattern was adhered onto PET substrate based on the mask‐assisted dry transfer method. During the fabrication of the cathode, a doped PPy/GO‐AM composite with admirable cyclability and flexibility has been electrodeposited on the flexible substrate. Significantly, a rGO/PAM protective polymeric layer was simultaneously self‐assembled and self‐adapted on Zn surface to improve the stability of the anode. The reversible Zn showed an outstanding cyclability for 1000 h at 1 mA cm^−2^/1 mAh cm^−2^. The obtained FZCs with these two optimized electrodes show excellent electrochemical and mechanical performance compared to other reported micro‐supercapacitors, including high capacitance, superior energy/power density, and favorable cyclability and bending flexibility. This novel manufacturing process presented here provides new insights into the fabrication of both the doped capacitive cathode and reversible Zn anode for high‐performance in‐plane flexible Zn‐based EESDs.

## Conflict of Interest

The authors declare no conflict of interest.

## Supporting information

Supporting Information

## Data Availability

The data that support the findings of this study are available from the corresponding author upon reasonable request.
