# Supplementary material for: Conformal Engineering of Both Electrodes Toward High‐Performance Flexible Quasi‐Solid‐State Zn‐Ion Micro‐Supercapacitors
Source: Adv Sci (Weinh). 2024 Apr 1;11(24):2308021. doi: 10.1002/advs.202308021 (PMC11200085; doi:10.1002/advs.202308021)
Supplement: Supplementary file 1 — Supporting Information [file ADVS-11-2308021-s001.pdf]

ADVANCED SCIENCE
Open Access

# Supporting Information

for *Adv. Sci.*, DOI 10.1002/advs.202308021

Conformal Engineering of Both Electrodes Toward High-Performance Flexible Quasi-Solid-State Zn-Ion Micro-Supercapacitors

*Yaopeng Wu, Wei Yuan*, Pei Wang, Xuyang Wu, Jinghong Chen, Yu Shi, Qianyi Ma, Dan Luo*, Zhongwei Chen* and Aiping Yu**

# Supporting Information

**Conformal Engineering of Both Electrodes toward High-Performance Flexible Quasi-Solid-State Zn-Ion Micro-Supercapacitors**

*Yaopeng Wu, Wei Yuan*, Pei Wang, Xuyang Wu, Jinghong Chen, Yu Shi, Qianyi Ma, Dan Luo*, Zhongwei Chen*, Aiping Yu**

Y. Wu, W. Yuan, P. Wang, X. Wu, J. Chen

School of Mechanical and Automotive Engineering, South China University of Technology, Guangzhou 510640, China

E-mail: mewyuan@scut.edu.cn

Y. Wu, Y. Shi, Q. Ma, D. Luo, Z. Chen, A. Yu

Department of Chemical Engineering, University of Waterloo, Waterloo N2L 3G1, Canada

E-mail: ld199257@gmail.com; zhwchen@uwaterloo.ca; aipingyu@uwaterloo.ca

## Experimental Section

*1. Materials*

Boric acid ($H_3BO_3$), zinc sulfate heptahydrate ($ZnSO_4{\cdot}7H_2O$), $\beta$-naphthalene sulfonic acid, pyrrole (Py), acrylamide (AM), potassium chloride (KCl), and gelatin were purchased from Sigma Aldrich. Graphene oxide (GO) was supplied by Nanjing MKNANO Tech Co., Ltd.

*2. Fabrication of flexible Zn-ion capacitors (FZCs)*

*Preparation of flexible interdigitated current collector film.* First, PET film (thickness: 12 μm) was engraved by UV laser (365 nm, 10 μm spot diameter, 20 W) to form an interdigitated ultra-thin mask. Then, PET substrate (thickness: 30 μm), ultra-thin mask, and commercial graphite sheet were stacked into a sandwich-like assembly. An interdigitated graphite current

collector (GCC) was directly dry-transferred onto the flexible PET substrate with assistance of pressure. Conductive silver paint was painted on the side of the current collectors for electrode preparation and electrochemical test.

*Electrodeposition of Zn anode.* The Zn anode was electrochemically deposited on one of the current collector with employing a constant current density of -20 mA $cm^{-2}$ for 900 s in a three-electrode system (GCC film, Ag/AgCl electrode and platinum plate were used as working, reference and counter electrodes, respectively). The aqueous electrolyte was prepared by dissolving 0.5 g of $H_3BO_3$, 3.125 g of $ZnSO_4{\cdot}7H_2O$, and 3.125 g of $Na_2SO_4$ in 50 mL of deionized water.

*Electrodeposition of PPy/GO-AM cathode.* The PPy/GO-AM cathode was electrochemically deposited on the other current collector with employing a constant current density of 1 mA $cm^{-2}$ for 1800 s in a three-electrode system (GCC film, Ag/AgCl electrode and platinum plate were used as working, reference and counter electrodes, respectively). The aqueous electrolyte was prepared by dissolving 0.565 g of $\beta$-naphthalene sulfonic acid, 0.335 g of Py, 0.033 g of GO, 0.5 g of AM in 50 mL of deionized water.

*Fabrication of rGO/PAM protective layer*. The rGO/PAM protective layer was deposited on Zn anode (rGO/PAM@Zn) through spontaneously reaction of GO and AM with Zn, during the electrodeposition of PPy/GO-AM cathode.

*Preparation of gel electrolyte*. The gel electrolyte was prepared by dissolving 1 g of gelatin, 0.745 g of KCl, and 2.875 g of $ZnSO_4{\cdot}7H_2O$ in 10 mL of deionized water, followed by stirring at 85 °C for 1h.

*Preparation of FZCs*. After the fabrication of PPy/GO-AM cathode and rGO/PAM@Zn anode, the device was rinsed several times with deionized water and ethanol. Afterwards, the device was placed in ambient at room temperature for 12 h to evaporate the residual deionized water and ethanol. Then, appropriate gel electrolyte was carefully drop-casted onto the surface

of the device. Finally, the FZC device was placed in ambient for 12 h to remove the redundant water in the gel electrolyte.

*3. Electrochemical measurements of PPy/GO-AM electrodes*

PPy/GO-AM electrode was fabricated by depositing PPy composite on graphite plate in the presence of GO and AM. In specific, graphite plate was cut into a rectangle, and insulated with tape to expose an area of 1 cm × 1 cm as the electrodeposition substrate. The PPy/GO-AM composite was electrochemically deposited on the graphite plate in the mentioned three-electrode system, where using the as-prepared graphite plate as working electrode. Flexible PPy/GO-AM on GCC electrode was also fabricated to investigate its cyclability and mechanical performance. Specifically, a rectangular GCC (area: 1 cm × 1 cm) was directly dry-transferred onto a PET substrate. Then, the flexible PPy/GO-AM electrode was fabricated in the mentioned three-electrodes system, where using the as-resulted PET substrate as working electrode. The electrochemical performance of PPy/GO-AM on graphite plate or GCC was measured in 1 M KCl solution in a three-electrodes system (PPy/GO-AM electrode, Ag/AgCl electrode and platinum plate were used as working, reference and counter electrodes, respectively). PPy on GCC electrode and PPy/GO on GCC electrode were also fabricated in the same way by changing the additives in the aqueous electrolyte. The CV curves at different scan rates, GCD cycles with different current density, and EIS under frequency ranging from 0.01 Hz to 100 kHz were measured by the PGSTAT320N Autolab electrochemical workstation. The flexibility tests were carried out with the assistance of a flexible electronics tester (FT1100, Prtronic).

*4. Electrochemical measurements of rGO/PAM@Zn electrodes*

rGO/PAM@Zn electrode was fabricated by placing Zn foil in GO/AM solution. In specific, Zn foil (diameter: 12 cm) was rinsed by dilute hydrochloric acid, and ground by sandpaper. Then, wipe clean the Zn foil, and place it in 10 mL GO/AM solution (6.6 mg of GO, 1 g of AM) for 1800 s. Take out the Zn foil, and rinse it several times with deionized water and ethanol. The rGO/PAM@Zn electrode was placed in ambient at room temperature for 24 h to evaporate

the residual deionized water and ethanol. The electrochemical performance was measured with CR2032 coin cell assembled by a glass fiber separator. The Zn plating/stripping performance was tested in Zn‖Zn (or rGO/PAM@Zn‖rGO/PAM@Zn) symmetrical cells in 2 M $ZnSO_4$ solution. The coulombic efficiency (CE) of Zn electrodes was tested with Cu‖Zn (or Cu‖rGO/PAM@Zn) cells at different current density. The Tafel plot was performed on a electrochemical station (CHI660E, China).

*5. Electrochemical measurements of FZCs*

The CV curves at different scan rates, GCD cycles with different current density were measured by the PGSTAT320N Autolab electrochemical workstation. The flexibility tests were carried out with the assistance of a flexible electronics tester (FT1100, Prtronic).

*6. Materials Characterizations.*

The morphologies, structures, and chemical analysis of the electrodes were characterized by scanning electron microscopy (SEM) (Merlin, Zesis), Raman spectroscopy (LabRAM Aramis, Horiba Jobin Yvon), X-ray diffraction (XRD) (X'pert Powder, PANalytical), and Fourier Transform Infrared spectrum (FTIR) (Nicolet IS50, Thermo Fisher Scientific). The wettability of electrodes was measured by a Contact Angle tester (CA, XG-CAMC33) using 2 uL of 1 M $ZnSO_4$ drop or 1 M KCl drop. Electron paramagnetic resonance (EPR) data were obtained using a Bruker spectrometer (EMXplus-6/1).

*7. Calculation*

The areal capacitance $C_A$ (F cm$^{-2}$), areal energy density $E_A$ (Wh cm$^{-3}$) and power density $P_A$ (W cm$^{-3}$) of the samples were calculated from CV and GCD response and the corresponding equations (1), (2), (3), (4), as follows:

$$C_A = \frac{1}{A_{electrode} \times v \times (V_{\max} - V_{\min})} \int_{V_{\min}}^{V_{\max}} i(V)dV \qquad (1)$$

$$C_A = \frac{I \times \Delta t}{V_{electrode} \times (V_{\max} - V_{\min})} \qquad (2)$$

$$E_A = \frac{1}{2} \times C_A \times \frac{(V_{max} - V_{min})^2}{3600} \quad (3)$$

$$P_A = \frac{3600 \times E_A}{\Delta t} \quad (4)$$

Among the four equations, $A_{electrode}$ is the area of active electrode materials. $v$ is the scan rate. $V_{max}$ and $V_{min}$ are the maximum and minimum output voltage of the CV curves. $i(V)$ is the voltammetry discharge current. $I$ is the charge-discharge current and $\Delta t$ refers to the discharge time in the GCD curves.

*8. Finite Element Simulation*

The COMSOL Multiphysics software was employed to theoretically simulate current and $Zn^{2+}$ ion concentration distribution on Zn anode during Zn plating process. A simplified 2D model was developed. The computational domain in this model has dimensions of 45 μm in length and 30 μm in height. To account for the uneven surface of the electrode, three bulges with a height of 5 μm and a length of 10 μm were strategically placed on the anode surface. These bulges were positioned 5 μm apart from each other. In terms of boundary conditions, a constant current density was applied to the anode side. On the other side of the computational domain, the boundary condition was set as “electrical ground”. rGO/PAM layer as a porous diffusion region is set at the height of 5 μm on the top of rGO/PAM@Zn.

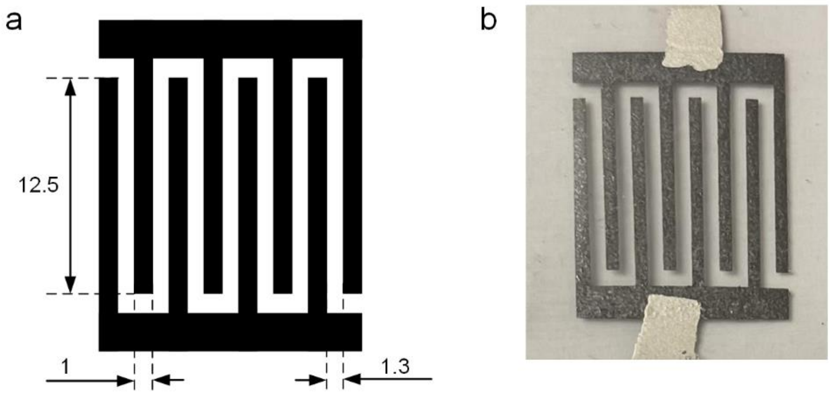

**Figure S1.** a) Geometrical parameters of interdigitated patterns for laser preparation of ultra-thin mask. b) The as-fabricated interdigitated GCC on flexible PET substrate.

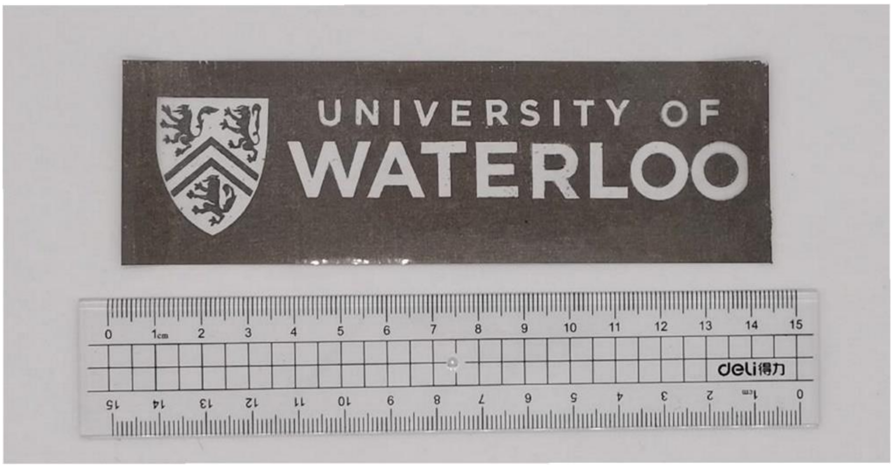


**Figure S2.** The photograph of GCC on flexible PET substrate with pattern of University of Waterloo logo prepared by mask-assisted dry transfer technology.

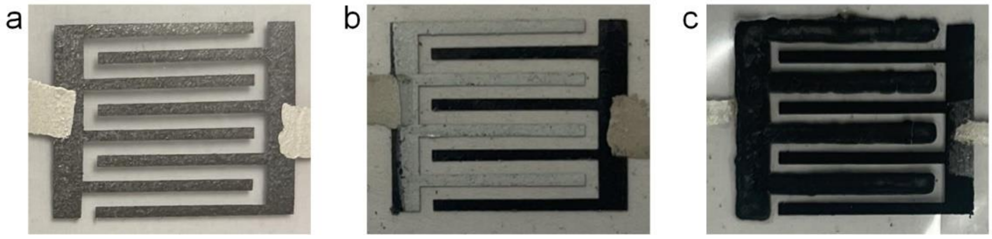


**Figure S3.** The photographs of sample in different stages during the preparation. a) Flexible PET substrate with interdigitated GCC layer. b) Zn deposited on one side of the GCC layer. c) PPy/GO-AM deposited on the other side of the GCC layer with rGO/PAM layer coated on Zn.

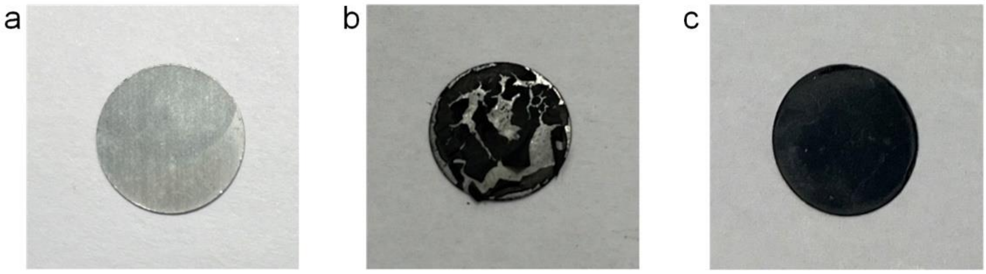


**Figure S4.** The photographs of Zn foil coated with different materials. a) Bare Zn. b) Zn foil with deposited rGO. c) Zn foil with deposited rGO/PAM.

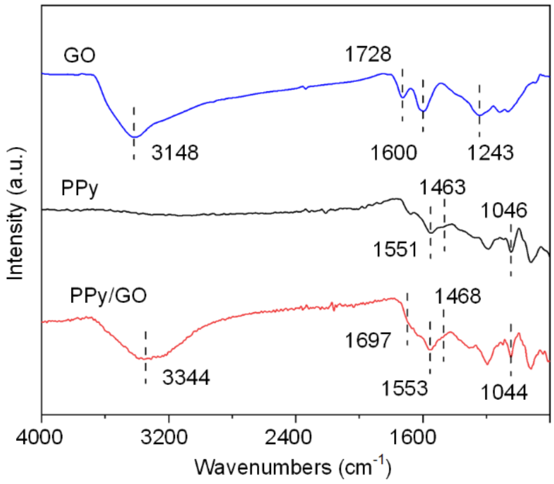


**Figure S5.** FTIR spectra of GO, PPy, and PPy/GO.

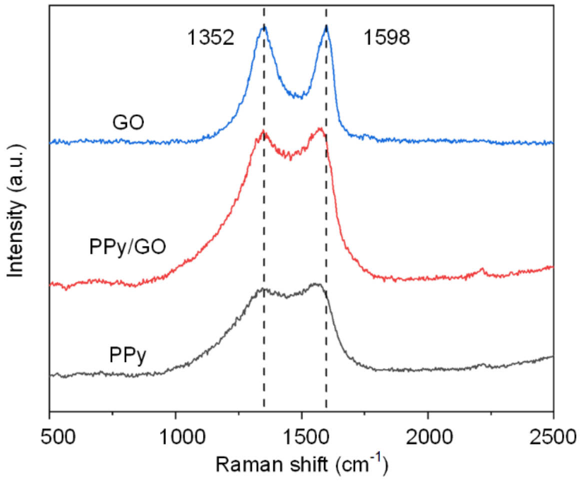


**Figure S6.** Raman spectra of GO, and PPy/GO.

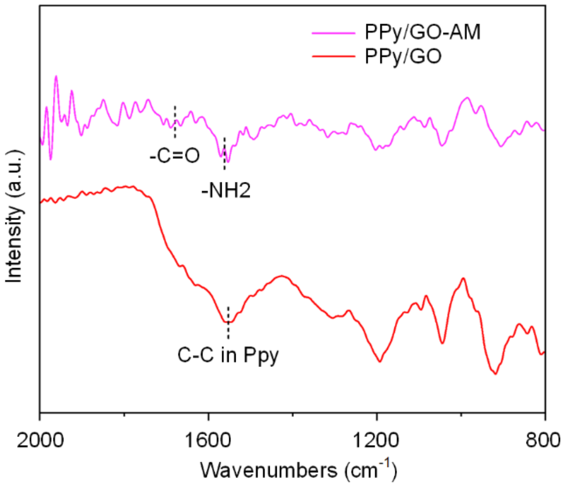


**Figure S7.** FTIR spectra of PPy/GO-AM and PPy/GO.

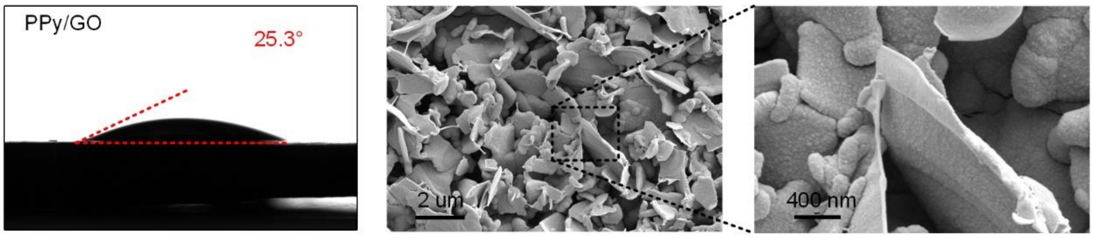


**Figure S8.** Wettability and SEM images of PPy/GO.

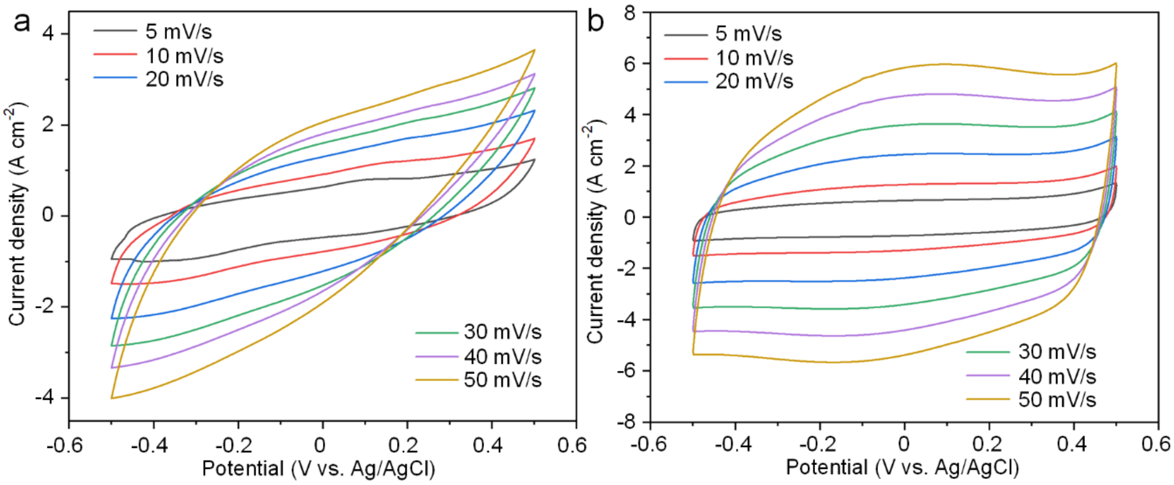


**Figure S9.** CV curves of (a) PPy and (b) PPy/GO at different scan rates.

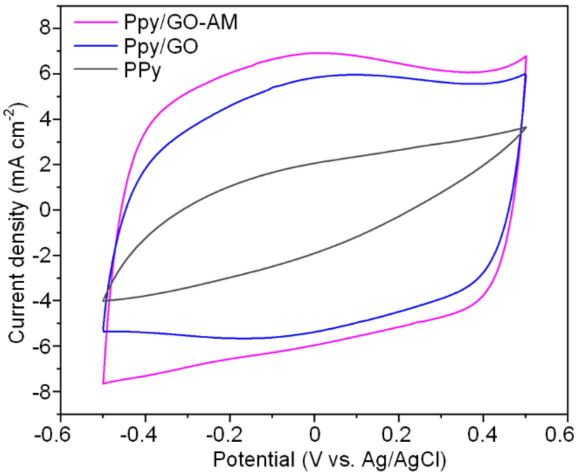


**Figure S10.** CV curves of PPy, PPy/GO, and PPy/GO-AM at scan rate of 50 mV $s^{-1}$.

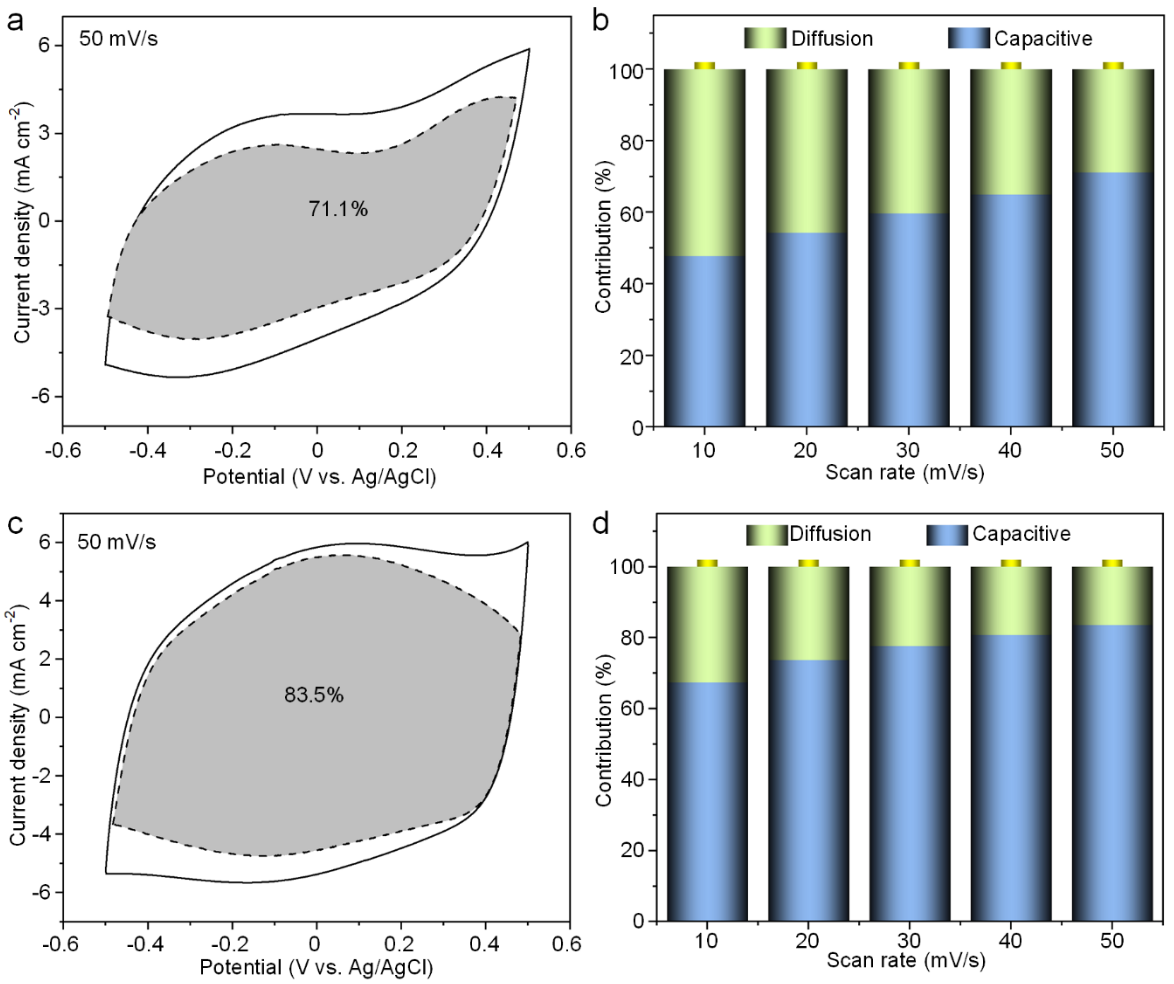


**Figure S11.** Capacitive contribution of (a) PPy, and (c) PPy/GO at scan rate of 50 mV $s^{-1}$. Capacitive ratios of (b) PPy, and (d) PPy/GO at stepwise scan rate.

**Table S1.** Calculated capacitance contributions of different materials at different scan rates.

| Samples | Scan rate (mV/s) | | | | |
|---|---|---|---|---|---|
| | 10 | 20 | 30 | 40 | 50 |
| PPy/GO-AM | 88.5 | 90.1 | 92.2 | 94.4 | 96.8 |
| PPy/GO | 67.3 | 73.7 | 77.6 | 80.8 | 83.5 |
| PPy | 47.7 | 54.8 | 59.7 | 65.0 | 71.1 |

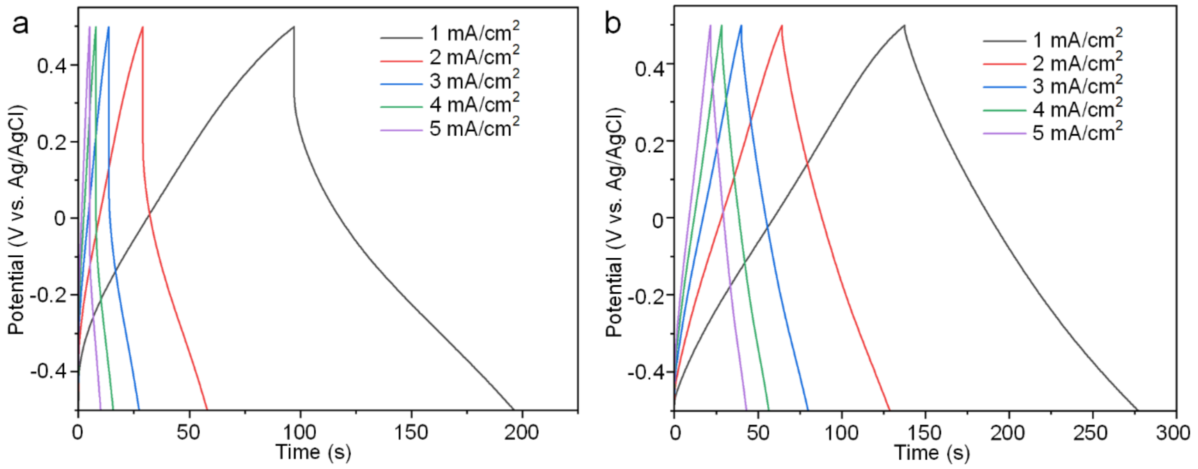


**Figure S12.** GCD curves of (a) PPy, and (b) PPy/GO at different current densities.

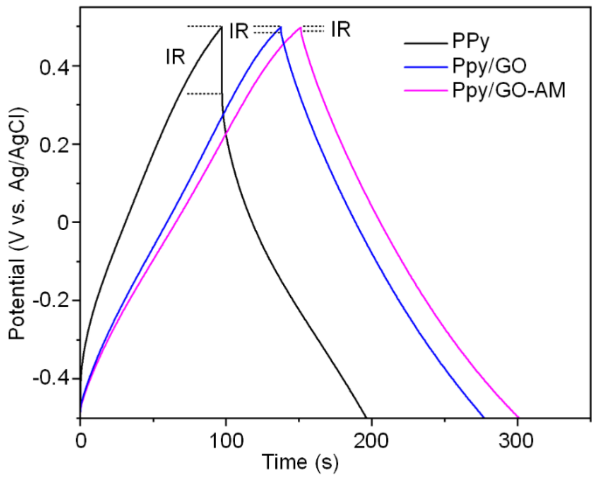


**Figure S13.** GCD curves of PPy, PPy/GO, and PPy/GO-AM at current density of 1 mA $cm^{-2}$.

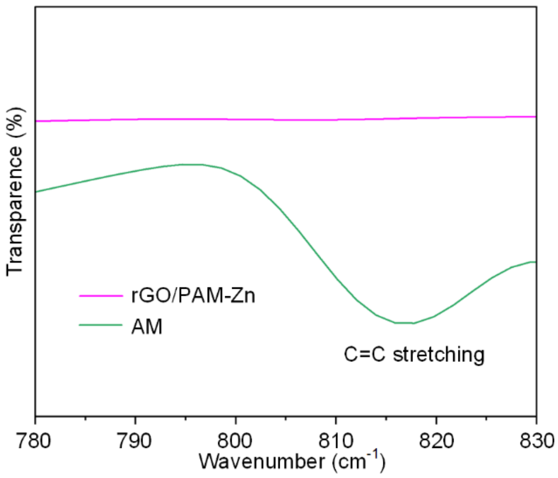


**Figure S14.** FTIR spectra of AM and rGO/PAM.

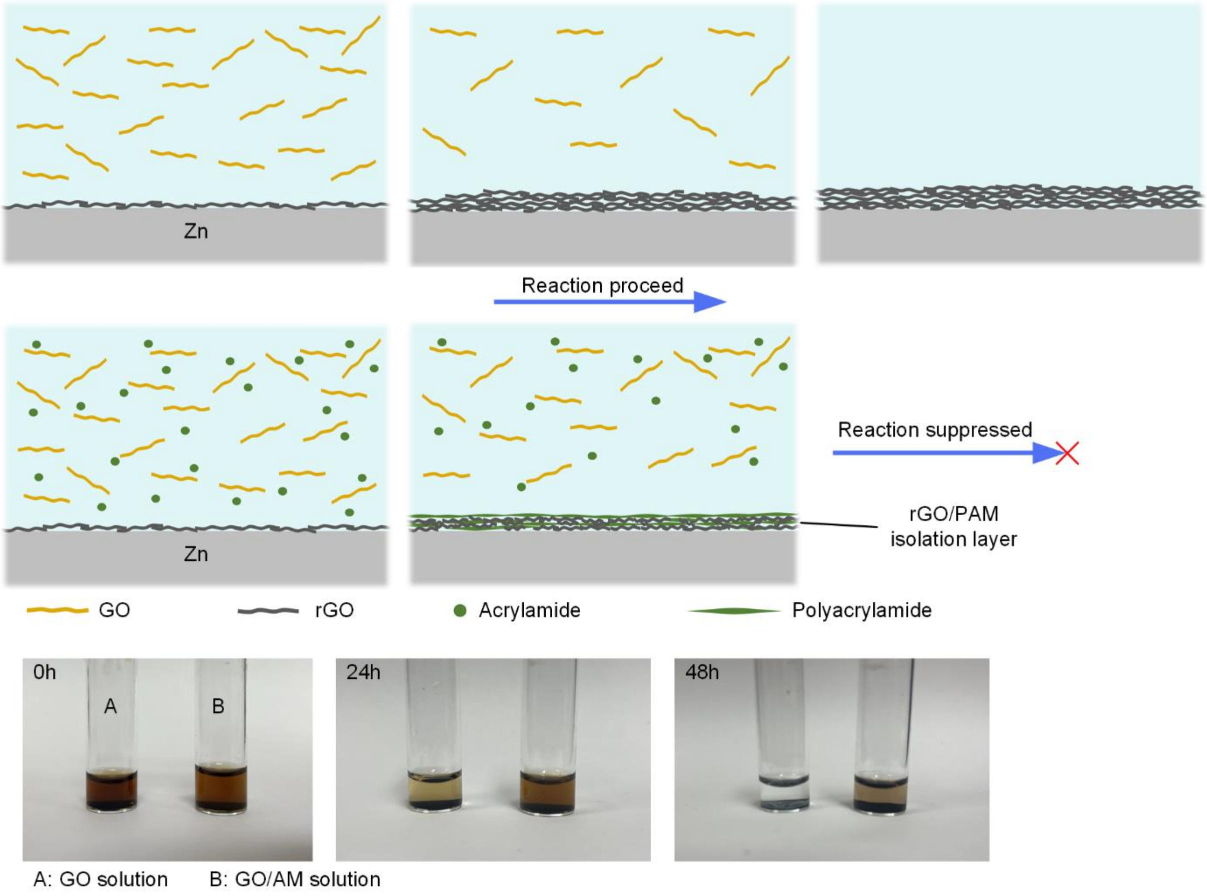


**Figure S15.** Schematic illustration of the reaction process between Zn and GO with or without AM additives, and the corresponding digital photos at different times.

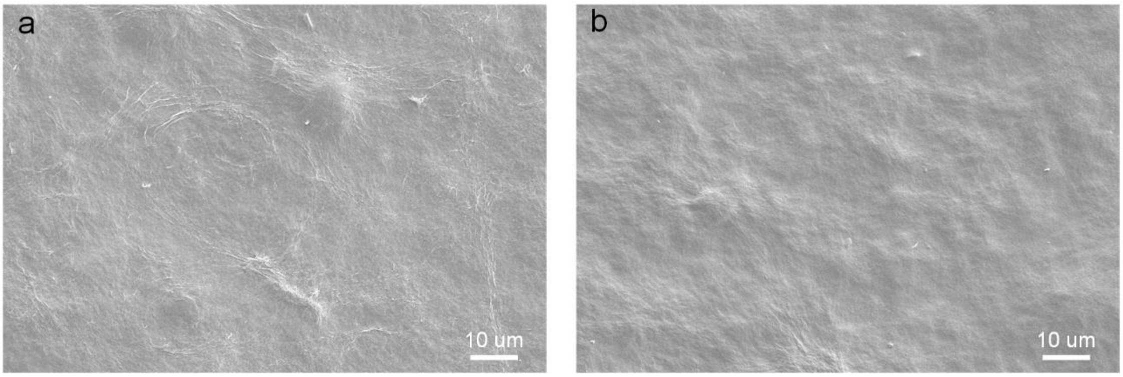


**Figure S16.** a) SEM images of rGO/PAM. b) SEM images of rGO/PAM(Py).

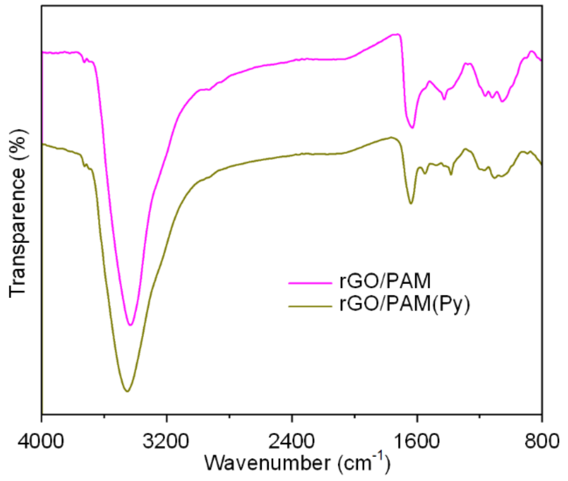


**Figure S17.** FTIR spectra of rGO/PAM and rGO/PAM(Py).

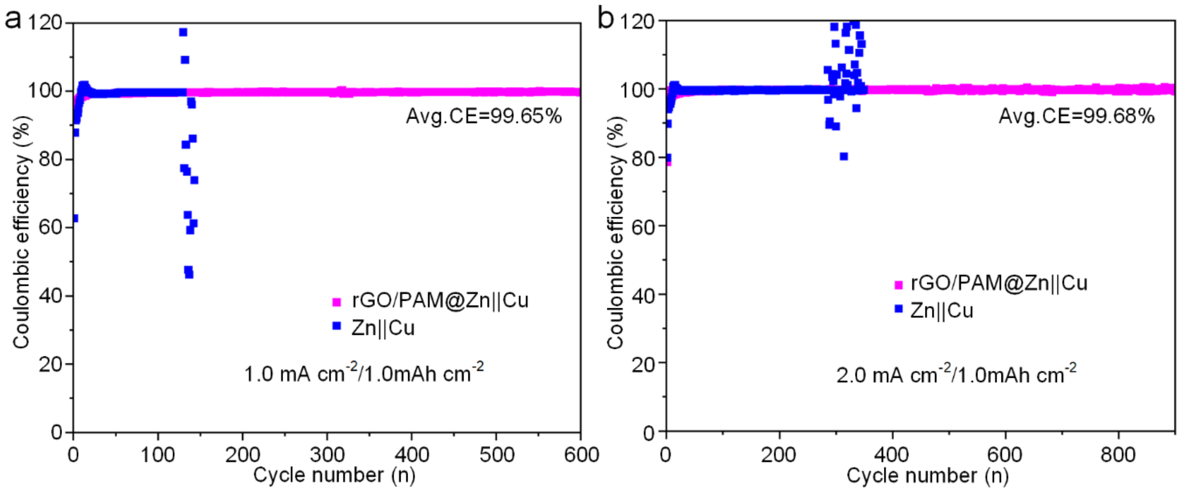


**Figure S18.** CE tests of Zn||Cu and rGO/PAM@Zn||Cu at a current density of (a) 1 mA $cm^{-2}$ and (b) 2 mA $cm^{-2}$.

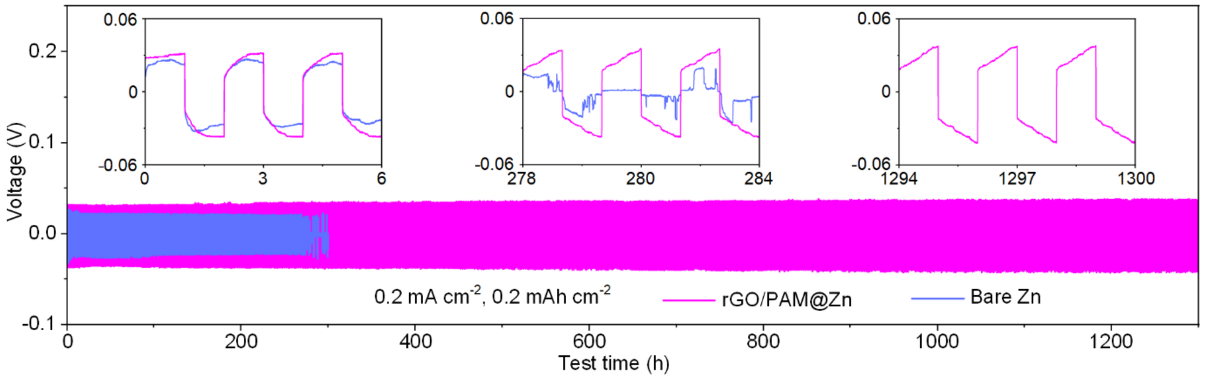

**Figure S19.** Long cycling performance of symmetrical bare Zn and rGO/PAM@Zn cell at a current density of 0.2 mA $cm^{-2}$.

**Table S2.** Comparison in cycling life between our rGO/PAM@Zn electrode and previously reported Zn electrodes on Zn symmetric cells.

| Zn anode | Preparation | Current density (mA $cm^{-2}$) | Areal capacity (mAh $cm^{-2}$) | Working time (h) | Ref. |
|---|---|---|---|---|---|
| rGO/PAM@Zn | In situ | 0.2<br>1 | 0.2<br>1 | 1300<br>1000 | This work |
| Sb@Zn | In situ | 1 | 1 | 800 | 1 |
| Sn@Zn | In situ | 1 | 1 | 500 | 2 |
| Zn-Sn alloy | In situ | 1 | 1 | 400 | 3 |
| $Sc_2O_3$@Zn | In situ | 1 | 1 | 200 | 4 |
| MXene@Zn | In situ | 0.2 | 0.2 | 800 | 5 |
| SC-PPS@Zn | Ex situ | 1 | 1 | 600 | 6 |
| CBL@Zn | Ex situ | 1 | 0.5 | 100 | 7 |
| PFSA@Zn | Ex situ | 1 | 1 | 800 | 8 |
| 502@Zn | Ex situ | 2 | 1 | 400 | 9 |
| G@Zn | Ex situ | 0.1 | 0.1 | 200 | 10 |
| 3DP@Zn | Ex situ | 0.5 | 1 | 800 | 11 |

MXene: two-dimensional transition metal carbides, carbonitrides and nitrides. SC-PPS: sulfonate-rich ion-exchange layer. CBL: chemical buffer layer. PFSA: perfluorosulfonic acid. 502: cyanoacrylate adhesive. G: pencil drawing graphite. 3DP: 3D printed stabilizer.

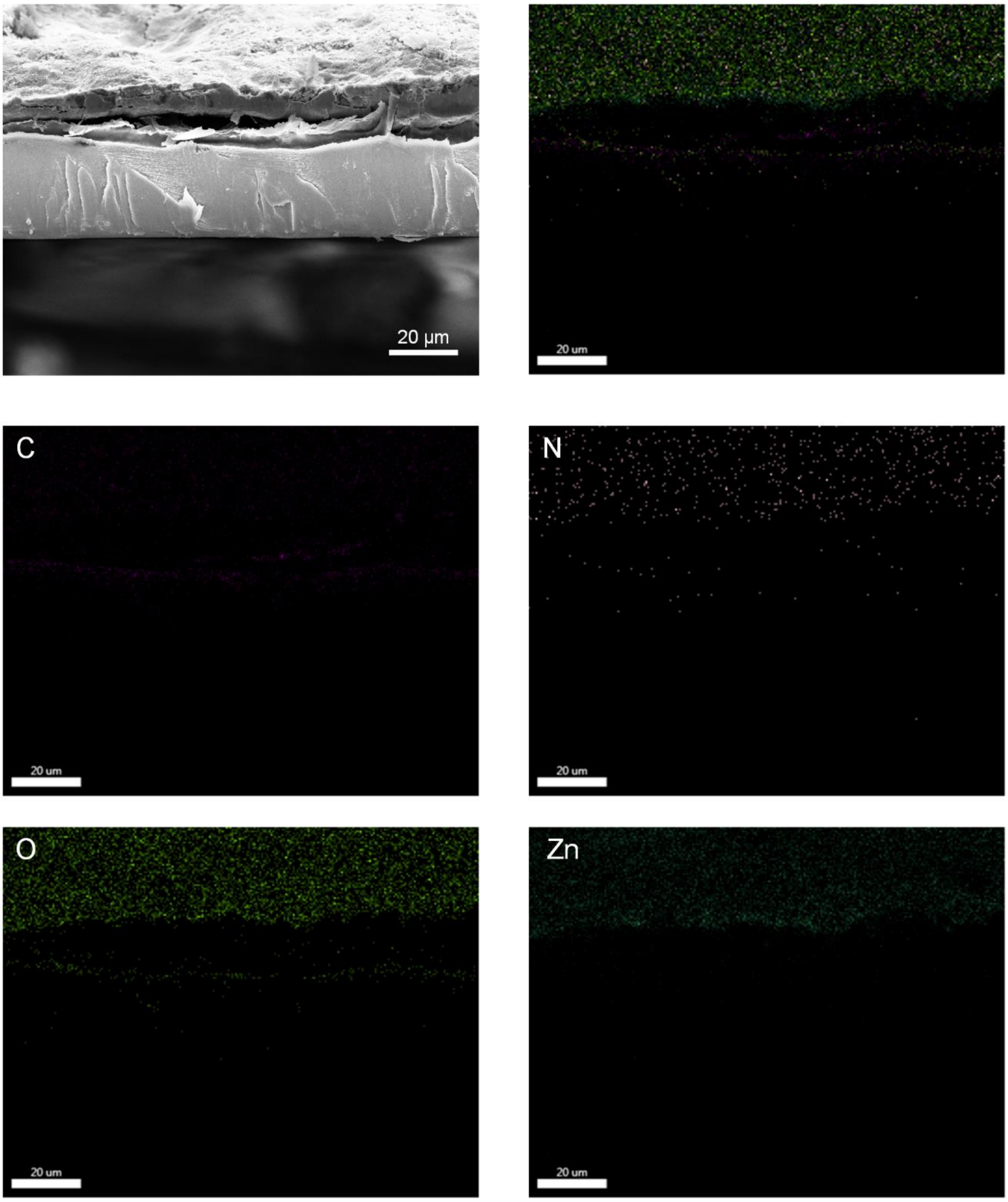


**Figure S20.** SEM images of flexible Zn anode (cross section), and corresponding elemental distribution of EDS mapping.

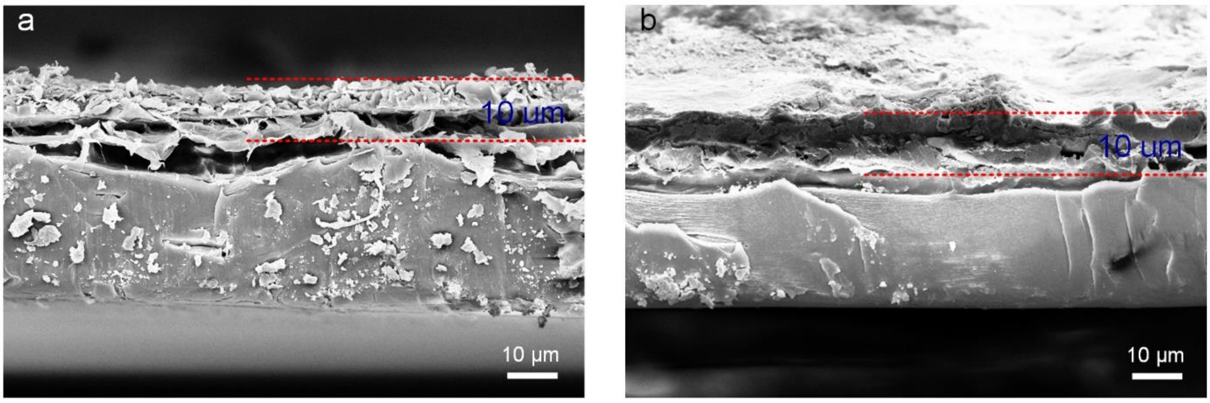


**Figure S21.** Cross sectional SEM images of flexible PPy/GO-AM cathode, and rGO/PAM@Zn anode.

**Table S3.** Performance comparison of our FZC device and previously reported Zn-based EESDs.

| Sample | Configuration | Potential window (V) | Capacitance (mF $cm^{-2}$) / (F $cm^{-3}$) | Energy density (μWh $cm^{-2}$) / (mWh $cm^{-3}$) | Power density (mW $cm^{-2}$) / (W $cm^{-3}$) | Cyclability | Ref. |
| --- | --- | --- | --- | --- | --- | --- | --- |
| rGO/PAM@Zn‖PPy/GO-AM | Flexible planar interdigitated | 0-1.6 | 125/125 (1 mA $cm^{-2}$) | 44.4/44.4 | 4.8/4.8 | 90.3% after 5000 (100 mV $s^{-1}$) | This work |
| Zn‖AC | Planar fixed | 0.5-1.5 | 900/112 (1 mA $cm^{-2}$) | 115.4/14.4 | 3.9/0.5 | 100% after 10000 (1.56 mA $cm^{-2}$) | 12 |

| | | | | | | | |
|---|---|---|---|---|---|---|---|
| Zn‖Mxene | Flexible planar interdigitated | 0-1.1 | 70/7.7 (1 mA $cm^{-2}$) | 7.53/0.83 | 4.4/0.48 | 86% after 5000 (3 mA $cm^{-2}$) | 13 |
| Zn‖Mxene | Flexible planar concentric circles | 0-1.4 | 50/588 (1 mA $cm^{-2}$) | 20/235 | 2.8/32 | 79.6% after 50000 | 14 |
| Zn‖CNT | Flexible planar interdigitated | 0.2-1.8 | 83/41.5 (1 mA $cm^{-2}$) | 29.6/14.8 | 8/4 | 87% after 6000 (3 mA $cm^{-2}$) | 15 |
| Zn‖MXene | Flexible braided coaxial structure | 0-1.2 | 112.4/- (1 mA $cm^{-2}$) | 20.23/- | 12.138/- | 84% after 6000 (3 mA $cm^{-2}$) | 16 |
| Zn‖Silicene | Coin cell | 0-1.8 | 10/- (1 mA $cm^{-2}$) | 5.5/- | 3/- | 112% after 1000 | 17 |
| Zn‖MXene/rGO | Flexible planar interdigitated | 0-1.35 | 65/- (1 mA $cm^{-2}$) | 10.1/- | 2/- | 81% after 1400 (2 mA $cm^{-2}$) | 18 |
| Zn‖kelp-C | Flexible planar | 0-1.7 | 7/7 | 8.2/8.2 | 4/4 | - | 19 |

| | | | | | | | |
|---|---|---|---|---|---|---|---|
| | interdigitated | | (1 mA $cm^{-2}$) | | | | |
| MXene\|\|MXene | Flexible planar interdigitated | 0-0.6 | 30/100 (0.8 mA $cm^{-2}$) | 2/6.6 | 0.25/0.83 | 100 after 300 | 20 |
| CuHCF/G\|\|FeHCF/G | Flexible planar interdigitated | 0-1.8 | 18.6/93 (5 mV $s^{-1}$) | 9.5/47.5 | 3.6/18 | 97% after 5000 (3 mA $cm^{-2}$) | 21 |
| VN\|\|$MnO_2$ | Flexible planar interdigitated | 0-2 | 12/28.9 (10 mV $s^{-1}$) | 9/21.6 | 0.6/1.4 | - | 22 |
| LIAG\|\|LIAG | Flexible planar interdigitated | 0.-0.8 | 25/- (0.5 mA $cm^{-2}$) | 4.01/- | 0.2/- | 95% after 6000 (0.2 mA $cm^{-2}$) | 23 |
| LIAG\|\|LIAG | Flexible planar interdigitated | 0-0.8 | 65/16.25 (1 mA $cm^{-2}$) | 11.4/2.85 | 0.4/0.1 | 96% after 5000 (0.2 mA $cm^{-2}$) | 24 |
| rGO-Ni\|\|rGO-Ni | Flexible planar interdigitated | 0-1.7 | 7/- (1 mA $cm^{-2}$) | 8.2/- | 4/- | - | 25 |

| | | | | | | | |
|---|---|---|---|---|---|---|---|
| $MnO_2$@PPy@CNT‖PPy@CNT | Flexible planar interdigitated | 0-1.6 | 21.8/54.4 (0.1 mA $cm^{-2}$) | 12/30 | 1.7/4.25 | - | 26 |

AC: active carbon. MXene: two-dimensional transition metal carbides, carbonitrides and nitrides. Silicene: 2D silicon nanosheets. Kelp-C: kelp derived carbon. CuHCF/G: alternately stacked graphene and Cu hexacyanoferrate layer. FeHCF/G: alternately stacked graphene and Fe hexacyanoferrate layer. VN: vanadium nitride. LIAG: laser-induced and KOH-activated graphene. rGO-Ni: rGO film coated on top of Ni-coated textile. PPy@CNT: co-electrodeposition of CNT and PPy on rGO current collector. $MnO_2$@PPy@CNT: electrodeposition of $MnO_2$ on PPy@CNT.

References

[1] L. Hong, L.-Y. Wang, Y. Wang, X. Wu, W. Huang, Y. Zhou, K.-X. Wang, J.-S. Chen, *Adv. Sci.* **2022**, *9*, 2104866.

[2] S. Li, J. Fu, G. Miao, S. Wang, W. Zhao, Z. Wu, Y. Zhang, X. Yang, *Adv. Mater.* **2021**, *33*, 2008424.

[3] L. Wang, W. Huang, W. Guo, Z. H. Guo, C. Chang, L. Gao, X. Pu, *Adv. Funct. Mater.* **2022**, *32*, 2108533.

[4] M. Zhou, S. Guo, G. Fang, H. Sun, X. Cao, J. Zhou, A. Pan, S. Liang, *J. Energy Chem.* **2021**, *55*, 549.

[5] N. Zhang, S. Huang, Z. Yuan, J. Zhu, Z. Zhao, Z. Niu, *Angew. Chemie Int. Ed.* **2021**, *60*, 2861.

[6] L. Zhang, J. Huang, H. Guo, L. Ge, Z. Tian, M. Zhang, J. Wang, G. He, T. Liu, J. Hofkens, D. J. L. Brett, F. Lai, *Adv. Energy Mater.* **2023**, *13*, 2203790.

[7] W. Sun, M. Ma, M. Zhu, K. Xu, T. Xu, Y. Zhu, Y. Qian, *Small* **2022**, *18*, 2106604.

[8] L. Hong, X. Wu, L.-Y. Wang, M. Zhong, P. Zhang, L. Jiang, W. Huang, Y. Wang, K.-X. Wang, J.-S. Chen, *ACS Nano* **2022**, *16*, 6906.

[9] Z. Cao, X. Zhu, D. Xu, P. Dong, M. O. L. Chee, X. Li, K. Zhu, M. Ye, J. Shen, *Energy Storage Mater.* **2021**, *36*, 132.

[10] Z. Li, L. Wu, S. Dong, T. Xu, S. Li, Y. An, J. Jiang, X. Zhang, *Adv. Funct. Mater.* **2021**, *31*, 2006495.

[11] Y. Liu, S. Zheng, J. Ma, X. Wang, L. Zhang, P. Das, K. Wang, Z.-S. Wu, *Adv. Energy Mater.* **2022**, *12*, 2200341.

[12] P. Zhang, Y. Li, G. Wang, F. Wang, S. Yang, F. Zhu, X. Zhuang, O. G. Schmidt, X. Feng, *Adv. Mater.* **2019**, *31*, 1.

[13] N. Wang, J. Liu, Y. Zhao, M. Hu, R. Qin, G. Shan, *ChemNanoMat* **2019**, *5*, 658.

[14] L. Li, W. Liu, K. Jiang, D. Chen, F. Qu, G. Shen, *Nano-Micro Lett.* **2021**, *13*, 100.

[15] G. Sun, H. Yang, G. Zhang, J. Gao, X. Jin, Y. Zhao, L. Jiang, L. Qu, *Energy Environ. Sci.* **2018**, *11*, 3367.

[16] B. Shi, L. Li, A. Chen, T.-C. Jen, X. Liu, G. Shen, *Nano-Micro Lett.* **2021**, *14*, 34.

[17] Q. Guo, J. Liu, C. Bai, N. Chen, L. Qu, *ACS Nano* **2021**, *15*, 16533.

[18] H. Zhang, Z. Wei, J. Wu, F. Cheng, Y. Ma, W. Liu, Y. Cheng, Y. Lin, N. Liu, Y. Gao, Y. Yue, *Energy Storage Mater.* **2022**, *50*, 444.

[19] J. Zeng, L. Dong, L. Sun, W. Wang, Y. Zhou, L. Wei, X. Guo, *Nano-Micro Lett.* **2020**, *13*, 19.

[20] P. Das, X. Shi, Q. Fu, Z.-S. Wu, *Adv. Funct. Mater.* **2020**, *30*, 1908758.

[21] Y. He, P. Zhang, M. Wang, F. Wang, D. Tan, Y. Li, X. Zhuang, F. Zhang, X. Feng, *Mater. Horizons* **2019**, *6*, 1041.

[22] J. Qin, S. Wang, F. Zhou, P. Das, S. Zheng, C. Sun, X. Bao, Z.-S. Wu, *Energy Storage Mater.* **2019**, *18*, 397.

[23] H. Liu, Y. Xie, J. Liu, K. Moon, L. Lu, Z. Lin, W. Yuan, C. Shen, X. Zang, L. Lin, Y. Tang, C.-P. Wong, *Chem. Eng. J.* **2020**, *393*, 124672.

[24] H. Liu, Y. Zheng, K.-S. Moon, Y. Chen, D. Shi, X. Chen, C.-P. Wong, *Nano Energy* **2022**, *94*, 106902.

[25] X. Pu, M. Liu, L. Li, S. Han, X. Li, C. Jiang, C. Du, J. Luo, W. Hu, Z. L. Wang, *Adv. Energy Mater.* **2016**, *6*, 1601254.

[26] J. Gao, C. Shao, S. Shao, F. Wan, C. Gao, Y. Zhao, L. Jiang, L. Qu, *Small* **2018**, *14*, 1801809.
